# Therapeutic Use of Transcranial Direct Current Stimulation in the Rehabilitation of Prolonged Disorders of Consciousness

**DOI:** 10.3389/fneur.2021.632572

**Published:** 2021-04-07

**Authors:** Davide Aloi, Antonio Incisa della Rocchetta, Alice Ditchfield, Sean Coulborn, Davinia Fernández-Espejo

**Affiliations:** ^1^School of Psychology, University of Birmingham, Birmingham, United Kingdom; ^2^Centre for Human Brain Health, University of Birmingham, Birmingham, United Kingdom; ^3^Acute Neurological Rehabilitation Unit, The Wellington Hospital, London, United Kingdom

**Keywords:** transcranial direct current stimulation, prolonged disorders of consciousness, traumatic brain injury, rehabilitation, electrophysiology

## Abstract

Patients with Prolonged Disorders of Consciousness (PDOC) have catastrophic disabilities and very complex needs for care. Therapeutic options are very limited, and patients often show little functional improvement over time. Neuroimaging studies have demonstrated that a significant number of PDOC patients retain a high level of cognitive functioning, and in some cases even awareness, and are simply unable to show this with their external behavior - a condition known as cognitive-motor dissociation (CMD). Despite vast implications for diagnosis, the discovery of covert cognition in PDOC patients is not typically associated with a more favorable prognosis, and the majority of patients will remain in a permanent state of low responsiveness. Recently, transcranial direct current stimulation (tDCS) has attracted attention as a potential therapeutic tool in PDOC. Research to date suggests that tDCS can lead to clinical improvements in patients with a minimally conscious state (MCS), especially when administered over multiple sessions. While promising, the outcomes of these studies have been highly inconsistent, partially due to small sample sizes, heterogeneous methodologies (in terms of both tDCS parameters and outcome measures), and limitations related to electrode placement and heterogeneity of brain damage inherent to PDOC. In addition, we argue that neuroimaging and electrophysiological assessments may serve as more sensitive biomarkers to identify changes after tDCS that are not yet apparent behaviorally. Finally, given the evidence that concurrent brain stimulation and physical therapy can enhance motor rehabilitation, we argue that future studies should focus on the integration of tDCS with conventional rehabilitation programmes from the subacute phase of care onwards, to ascertain whether any synergies exist.

## Introduction

Prolonged Disorders of consciousness (PDOC) are heterogeneous medical conditions characterized by absent or limited awareness for at least 4 weeks after severe brain injury (1). Therefore, PDOC refers specifically to the vegetative (VS, also called unresponsive wakefulness syndrome) and minimally conscious states (MCS, including those emerging from this condition) (2), and excludes acute conditions such as coma or cases where diagnosis has not been confirmed yet. PDOC diagnosis is based on clinical criteria, whereby the MCS differs from the VS by the fluctuating presence of reproducible behaviors that can be associated with awareness of the patient's self or of their environment. This includes the ability to follow simple commands, gestural or verbal yes/no responses, intelligible verbalisation, or some purposeful behaviors [see (3)]. These behaviors are not present in the VS, and patients are assumed to be entirely unaware of themselves and their environment as a result. Importantly, neuroimaging studies have demonstrated that a significant number of PDOC patients retain a high level of cognitive functioning, and in some cases awareness, but are unable to show this with their external behavior. This condition has been recently named cognitive-motor dissociation [CMD; (4)], and is typically revealed by the ability to follow commands in functional magnetic resonance (fMRI) or electroencephalography (EEG) motor imagery tasks. The discovery of covert command-following in some PDOC patients has wide implications for diagnosis and is sometimes associated with a more favorable prognosis. Specifically, recent evidence has shown that, when evident in the early stages [i.e., acutely, (5, 6); or within the first year post injury, (7)], CMD may be a good predictor for a better outcome. In Edlow et al. (5), three out of four patients identified with CMD recovered beyond a confusional state by 6 months (one died), as measured by the Glasgow Outcome Scale-Extended (GOSE). Similarly, Pan et al. (7) reported that 15 out of 18 VS patients with CMD regained consciousness (compared to 5 out of 27 in a true VS) and 14 out of 16 MCS patients with CMD improved their CRS-R scores (compared to only 4 out of 17 MCS patients without CMD), at a 3-month follow-up. Finally, in a large study including 141 patients, Johr et al. (6) showed that patients presenting with potential clinical CMD (as assessed by the revised Motor Behavior Tool; 8) exhibited much more favorable recovery trajectories across cognitive and functional domains than their PDOC counterparts.

Despite these encouraging possibilities, therapeutic options for PDOC patients are still very limited, and many patients show little functional improvement over time, or even progress to a permanent state of low responsiveness. In fact, national guidelines of care proposed by the Royal College of Physicians (1) do not recommend active rehabilitation in PDOC, but advise instead disability management, maintenance therapy, and responsiveness monitoring. Recently, transcranial direct current stimulation (tDCS) has attracted attention as a potential non-pharmacological rehabilitative tool for treatment of patients with PDOC. tDCS is a non-invasive brain stimulation technique that works by delivering a direct current from a stimulator through electrodes placed on the scalp, with the aim of modulating brain activity (8). When electric current reaches the brain, it creates an electric field that polarizes or depolarises underlying neurons based on the field's magnitude and direction (8). These changes can impact cortical excitability and plasticity and influence cognitive functions as a result (8). Traditionally, tDCS was thought to affect only cortical areas. This would limit its application in PDOC, where subcortical and thalamo-cortical networks are known to play a crucial role (9). However, there is now growing evidence from both animal (10) and human studies (11–13), that tDCS can indirectly influence deeper structures too, when these are functionally connected to the cortical target region in a relevant cognitive function. In line with this, we have found evidence that anodal tDCS over the motor cortex and cathodal tDCS over the cerebellum lead to long-range changes in thalamo-cortical coupling when participants are engaged in a motor task (14), and therefore has potential to target the specific mechanisms underlying CMD (15). Moreover, as discussed elsewhere (16), modeling and intra-cranial recordings have also shown that tDCS can also generate significant current in deeper regions to expect successful neuromodulations (17–22). Importantly, tDCS is associated with a low risk to induce seizures and is highly portable and easy to deliver, which makes it a particularly attractive option for PDOC patients, whether they are hospitalized or treated at home.

Several studies have reported short-term improvements in behavioral responsiveness of PDOC patients after tDCS (17, 23–31). While very promising, their outcomes have sometimes been inconsistent, partially due to small sample sizes, heterogeneous methodologies across studies (e.g., differences in montage, current intensity, or target area, amongst others) and variability in the outcome measures used (from behavioral scales to electroencephalographic recordings).

Here, we review findings in support of the use of tDCS as an intervention to support rehabilitation in PDOC, with a specific focus on CMD patients. We identify several areas for consideration when interpreting the outcomes of current literature of tDCS on PDOC and propose ways to address these to increase robustness of conclusions in future research. We also review whether the use of neuroimaging data can help identify tDCS changes that are not behaviorally evident. Finally, we argue that tDCS can be integrated with routine care programmes to maximize its beneficial effects.

## Clinical Effects of tDCS in PDOC

A total of 20 studies have investigated the effects of tDCS stimulation in the level of consciousness of PDOC patients. Among these, nine considered effects of a single-session of tDCS (23, 25, 32–38) and the remaining 11 involved multiple tDCS sessions (5–20, 24, 26–30, 39–43) (see Tables 1, 2). While the majority of the studies used a double-blind, sham-controlled randomized crossover design, a small number used (double-blind, sham-controlled) randomized non-crossover designs (27, 42), open-label sham-controlled designs (24, 34), or open-label without a sham condition (29, 30, 43). All used anodal stimulation as the active condition. The duration of the stimulation was typically around 20 min, at amplitudes between 1–2 mA. Sample sizes ranged from 10 to 45 patients and included either MCS only or both VS and MCS patients.

**Table 1:** Overview of single session tDCS studies in PDOC.

**References**	**Patients**	**Type of study**	**tDCS montage**	**Stimulation parameters (intensity, electrode size, duration)**	**Immediate post-stimulation evaluations**	**Follow up**	**Dependant measure**	**tDCS elicited changes**
Thibaut et al. (23)	25 VS, 30 MCS.	Double-blind sham-controlled randomized crossover.	Anode: left DLPFC; Cathode: right supraorbital region.	1 mA, 35 cm^2^, 20 min	Immediately post-stimulation.	12 months.	CRS-R.	• Increased CRS-R scores in MCS only. • At the individual level, 13 MCS patients and 2 VS patients showed signs of consciousness which were not observed before active tDCS nor after sham tDCS.
Naro et al. (32)	20 HC, 10 VS, 12 MCS, 2 EMCS, 1 LIS.	Double-blind sham-controlled randomized crossover.	Anode: OFC; Cathode: Cz.	1 mA, 25 cm^2^ (Anode), 35 cm^2^ (Cathode), 10 min.	Immediately and 60' post-stimulation.	–	CRS-R, TMS and MEPs.	• No effects on CRS-R scores in any clinical group • Increased cortical excitability in HC and MCS patients • Increased M1 excitability and premotor-motor connectivity in 4 VS patients (no group effects).
Naro et al. (25)	10 HC, 10 VS, 10 MCS.	Double-blind sham-controlled randomized crossover.	Anode: medial cerebellum; Cathode: left buccinators muscle.	Oscillatory tDCS: rectangular waves changing polarity at 5 Hz, between 0 and 2 mA per electrode, 16 cm^2^, 10 min.	Immediately, after 30' and after 60' post-stimulation.	–	CRS-R, EEG: absolute power spectra (POW), coherence (COH).	• Increased CRS-R scores in MCS only. • Increased absolute power spectra and coherence in the fronto-parietal network increased in HC and MCS.
Bai et al. (33)	9 VS, 7 MCS.	Double-blind sham-controlled randomized crossover.	Anode: left DLPFC; Cathode: right supraorbital.	2 mA, 25 cm^2^, 20 min.	Immediately post-stimulation	–	TMS-EEG (global mean field amplitude).	• Changes in excitability in temporal and spatial domains that are different between MCS and VS.
Bai et al. (34)	9 VS, 8 MCS.	Sham controlled crossover.	Anode: L-DLPCF; Cathode: right supraorbital area.	2 mA, 25 cm^2^, 20 min.	Immediately post-stimulation	–	CRS-R, EEG: coherence.	• No effects in CRS-R scores • Modulations of fronto-parietal coherence in MCS: increase in theta band and decrease in gamma band coherence.
Martens et al. (35)	4 VS, 6 MCS.	Double-blind, sham-controlled randomized crossover trial study.	Anodal: M1 (C3 or C4); Cathode: supraorbital area (contralateral).	2 mA, 35 cm^2^, 20 min.	Immediately post-stimulation	–	CRS-R.	• No effects in CRS-R scores.
Thibaut et al. (36)	5 VS, 7 MCS, 1 MCS+,1 LIS	Double-blind, sham-controlled randomized crossover pilot study.	Multichannel: 2 Anodes: L/R DLPFC; 2 Cathodes: L/R M1.	1 mA, 20 min.	Immediately post-stimulation	–	MAS, CRS-R, EEG: relative power band and wPLI.	• No effects in CRS-R scores. Four patients (1 VS, 2 MCS and 1 EMCS) showed reduced spasticity in finger flexors although no group level effect was observed. • Increased connectivity (wPLI) values in beta-2 EEG band, higher relative power in theta band, and higher connectivity in beta band in responders.
Carrière et al. (37)	13 MCS /MCS+.	Double-blind, sham-controlled randomized crossover pilot study.	Anode: L-DLPFC; Cathode: right supraorbital region.	1 mA, 35 cm^2^, 20 min.	Immediately post-stimulation	–	CRS-R, HD-EEG: connectivity (wPLI, wSMI) and power.	• No effects in CRS-R scores. Higher relative power l in the alpha band (central regions) and theta band (frontal and posterior regions). Higher wSMI connectivity between left and right parietal regions, and higher fronto-parietal wPLI. • NB. both EEG effects are uncorrected for multiple comparisons.
Martens et al. (38)	17 VS, 23 MCS, 6 MCS+.	Double-blind sham-controlled randomized crossover trial.	Multichannel: Anodes: F3, F4, CP5 and CP6 (bilateral fronto-parietal areas); Cathode: FP2, FPz (prefrontal) and O1, Oz (occipital).	1 mA per anode, 3.14 cm^2^, 20 min.	10' post-stimulation.	–	CRS-R, EEG: relative band power and LZW complexity.	• No effects in CRS-R scores. • Increase in EEG complexity for low-frequency bands (1–8 Hz). • Changes in CRS-R scores negatively correlated with LZW complexity for those bands.

*CRS-R, Coma recovery scale revised; DLPFC, dorsolateral prefrontal cortex; EEG, electroencephalography; EMCS, exit-minimally conscious state; HC, healthy controls; HD-EEG, high-definition EEG; LIS, locked-in syndrome; LZW, Lempel-Ziv-Welch; M1, motor cortex; MAS, Modified Ashworth Scale; MCS, minimally conscious state; MCS+, high-level MCS; OFC, orbitofrontal cortex; VS, vegetative state; wPLI, debiased weighted phase lag index*.

**Table 2:** Overview of multiple session tDCS studies in PDOC.

**References**	**Patients**	**Type of study**	**tDCS montage**	**Stimulation parameters (intensity, electrode size, duration)**	**Number of sessions**	**Immediate post-stimulation evaluations**	**Follow up**	**Dependant measure**	**tDCS elicited changes**
Angelakis et al. (24)	7 VS, 3 MCS.	Open-label, sham-controlled, case series trials.	Anode: L-DLPFC or L-M1; Cathode: right orbit.	1/2 mA, 25 cm^2^, 20 min.	15 (5 1 mA, 5 2 mA, 5 sham).	Immediately after each week's last stimulation session.	12 months	CRS-R.	• CRS-R scores increased in 3 MCS patients and 1 VS patient. The VS patient who showed a clinical improvement was diagnosed as MCS- at a 12-month follow-up.
Estraneo et al. (40)	7 VS, 6 MCS.	Double-blind sham-controlled randomized crossover.	Anode: L-DLPFC; Cathode: right supraorbital region.	2 mA, 35 cm^2^, 20 min.	20 (5 anodal, 5 sham).	Immediately after the first stimulation session and 2 h after the last weekly stimulation.	3 months	CRS-R, EEG: background activity (visual classification by 2 clinicians).	• No effects in CRS-R scores. • Some MCS patients with short time post-injury showed small increases in CRS-R total score. • EEG changes occurred in conjunction with small clinical improvements.
Thibaut et al. (26)	16 MCS.	Double-blind sham-controlled randomized crossover.	Anode L-DLPFC; Cathode: right supraorbital region.	2 mA, 35 cm^2^, 20 min.	10 (5 anodal, 5 sham).	Immediately post-stimulation in all sessions	1 week	CRS-R.	• CRS-R scores increased in chronic MCS patients. The effects were present both at day 5 as well as 1 week after the last stimulation.
Huang et al. (17)	33 MCS.	Double-blind sham-controlled randomized crossover.	Anode: posterior parietal cortex (Pz). Cathode: right supraorbital region.	2 mA, 20 min.	10 (5 anodal, 5 sham).	Immediately post-stimulation in all sessions	5 days	CRS-R.	• CRS-R scores increased in 9 MCS patients.
Zhang et al. (27)	real tDCS: 5 VS, 8 MCS. Sham tDCS: 6 VS and 7 MCS.	Double-blind sham-controlled randomized, non-crossover.	Anode: L-DLPFC; Cathode: right supraorbital region.	2 mA, 35 cm^2^, 20 min.	20, twice a day for 10 days.	Immediately after last stimulation session for each condition	Directly after	CRS-R, EEG: auditory odd-ball paradigm, amplitude and latency (auditory ERP, p300).	• Increased CRS-R scores increased in MCS only. • Significant increase in P300 amplitude (but not in latency) in MCS patients.
Martens et al. (28)	22 MCS.	Double-blind sham-controlled randomized crossover.	Anode: L-DLPFC; Cathode: right supraorbitalregion. Cathode right supraorbital region	2 mA, 35 cm^2^, 20 min.	40 (20 anodal, 20 sham) for 4 weeks.	Immediately after last stimulation session for each condition	3 months	CRS-R.	• No group effects on CRS-R scores. • At the individual level, six patients showed new signs of consciousness at the end of the 20 sessions of active tDCS, that were not present after the sham sessions. • No improvements present at a 12-month follow-up.
Cavinato et al. (41)	12 VS, 12 MCS.	Double-blind sham-controlled randomized crossover.	Anode: L-DLPFC; Cathode: contralateral deltoid.	2mA, 35cm^2^, 20 min.	20 (10 anodal over 2 weeks, 10 sham over 2 weeks).	Immediately after last stimulation session for each condition.	Directly after	CRS-R, WNSSP, EEG: coherence and power spectra.	• No effects on CRS-R scores. MCS patients showed an increase in WNSSP score, in power and coherence in frontal and parietal EEG bands. VS patients showed an increase in frontal coherence in the delta band but no changes in power spectra.
Straudi et al. (29)	10 MCS.	Open-label pilot study.	2 anodes: bilateral M1; Cathode: Nasion.	2 mA, 16 cm^2^, 40 min.	10 over 2 weeks.	Immediately after each week's last stimulation session	3 months	CRS-R, EEG: relative band power.	• Increased CRS-R scores in 8 out of 10 MCS patients. • In MCS patients, the alpha upper band at the parietal site was increased after 5 stimulation sessions out of 10.
Wu et al. (42)	9 VS, 7 MCS.	Double-blind sham-controlled randomized, non-crossover.	Anode: L-DLPFC or R-DLPFC; Cathode: corresponding contralateral supraorbital area.	2 mA, 35 cm^2^, 20 min.	10 anodal or sham over 2 weeks.	Immediately after each week's last stimulation session.	3 months	CRS-R, GOS-E, EEG: connectivity (PLV).	• No effects on CRS-R scores. • L-DLPFC tDCS led to increased excitability of the prefrontal cortex. • R-DLPFC tDCS only elicited activation in the right frontal lobe and no increases in region-to-region connections.
Guo et al. (43)	5 VS, 6 MCS.	Open-label pilot study.	HD-tDCS (4x1-ring) Anode: 1 electrode over the precuneus; Cathode: 4 electrodes surrounding the anode (each placed 3.5 cm radially from the anode).	2 mA, 20 min.	28 anodal over 14 consecutive days (twice a day).	Immediately after the 2nd, 14th, and last sessions.	–	CRS-R, EEG: coherence.	• Increased CRS-R scores in all MCS patients and 3 VS patients. • Reduced coherence in the delta band, for frontal inter-hemisphere, central inter-hemisphere and between the central and parietal region.
Zhang et al. (30)	15 VS, 20 MCS.	Open-label study.	HD-tDCS (4x1-ring) Anode: 1 electrode over the precuneus; Cathode: 4 electrodes surrounding the anode (each placed 3.5 cm radially from the anode).	2 mA, 20 min.	28 anodal over 14 consecutive days (twice a day).	Immediately after the 2nd, 14th, and last sessions.	–	CRS-R; EEG: clustering coefficient and global efficiency based on dwPLI.	• Increased CRS-R scores in 11 MCS patients and 5 VS patients. • Increased average clustering coefficient values in the beta and gamma bands for MCS patients only. • Decreased global efficiency values in the delta band and increased values in the beta band for MCS patients only.

*CRS-R, Coma recovery scale revised; DLPFC, dorsolateral prefrontal cortex; dwPLI: debiased weighted phase lag index; EEG, electroencephalography; ERP, event-related potential; GOS-E, Glasgow Outcome Scale-extended; M1, motor cortex; MCS, minimally conscious state; PLV, phase-locking value; VS, vegetative state; WNSSP, Western Neuro Sensory Stimulation Profile*.

### Target Areas

**Figure 1:**
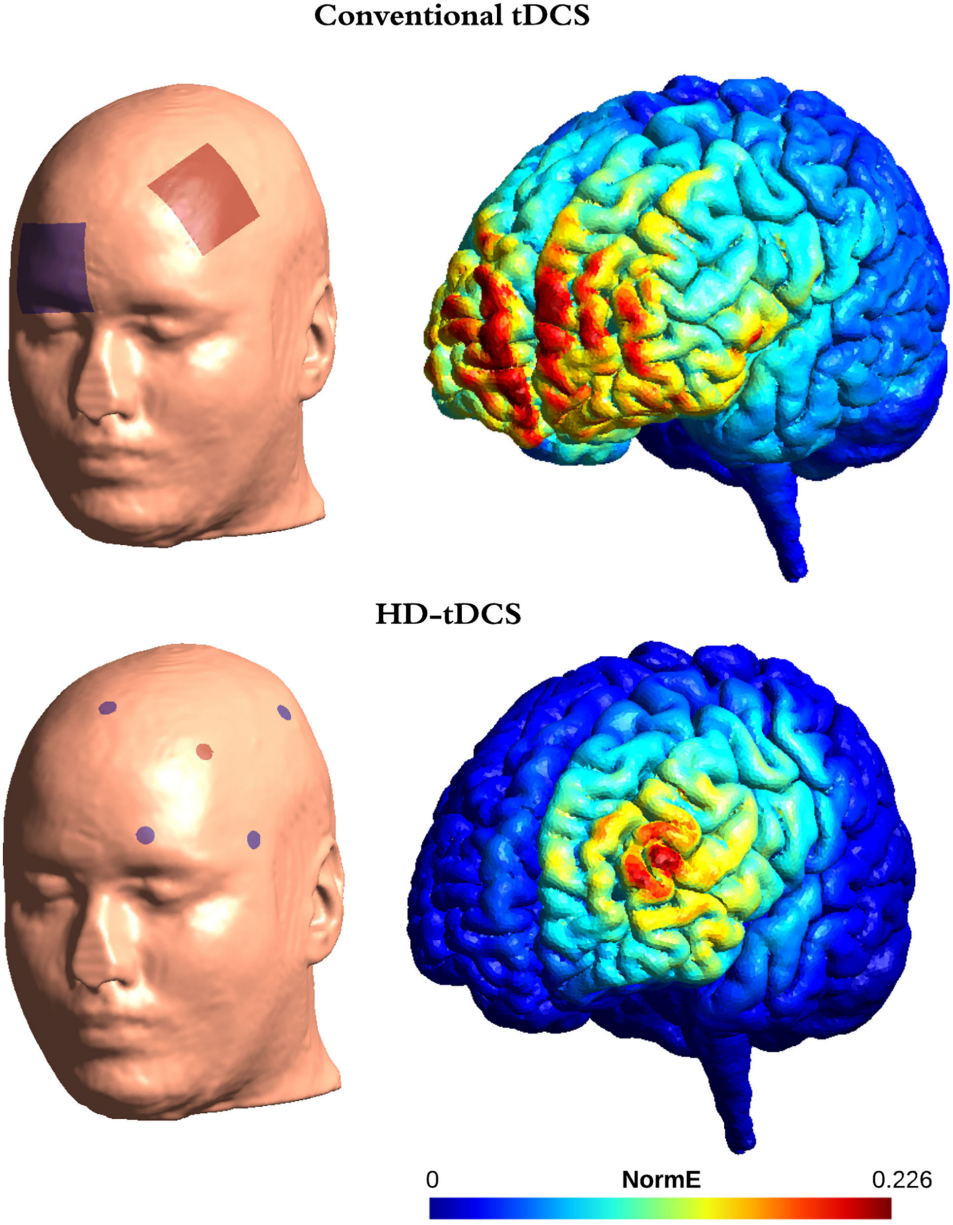
Comparison between conventional tDCS and HD-tDCS montages targeting the left DLPFC. The top two inset displays the most commonly used montage to target the DLFC with conventional tDCS: active electrode (anode) placed over F3 and the reference electrode (cathode) over Fp2. The bottom two insets display the equivalent HD-tDCS montage: active electrode over F3 and 4 reference electrodes, placed at FP1, FZ, C3 and F7. For each montage, the left inset represents the location of each electrode on a three-dimensional head model and the right inset represents the simulated electric field on a standard brain. Red colors represent higher fields. We used Simnibs3.2.2 on the “Ernie” head model with the current intensity for the active electrode set at 1 mA.

The most commonly targeted area was the left DLPFC (12 studies; 21, 22, 24–26, 30, 31, 33, 34, 36–38). A small number of studies investigated other areas such as the motor cortex (24, 29, 35), cerebellum (25), right DLPFC (36, 42), orbitofrontal cortex (OFC) (32), posterior parietal cortex (17), or bilateral fronto-parietal areas (38). In addition to these standard tDCS montages, two studies (30, 43) applied a more recent method known as in high-definition tDCS (HD-tDCS) over the precuneus, for 14 consecutive days. See Figure 1 for an example of a canonical and a HD-tDCS montage.

All studies except Bai et al. (33) used the Coma Recovery Scale Revised [CRS-R; (44)] as one their main outcome measures. This is currently the gold-standard for diagnosis in PDOC. Out of these 19 studies, 10 did not report significant changes in behavioral responsiveness at the group level following tDCS (28, 32, 34–38, 40–42). Interestingly however, eight of these reported significant EEG changes (see section below).

In contrast, nine studies reported significant changes in CRS-R scores after tDCS, although those changes were limited to MCS patients only [with the exception of Zhang et al. (30) who also observed increased CRS-R scores in VS patients, although this increase was significantly lower to the one observed in MCS patients]. Among these studies, three targeted the left dorsolateral prefrontal cortex (L-DLPFC) (23, 26, 27), one the cerebellum (25), one targeted both the primary motor cortex (M1) and the L-DLPFC (24), one bilateral M1 (29), one the posterior parietal cortex (17), and two the precuneus (30, 43).

### Number of Treatment Sessions

Seven of the nine studies reporting clinical changes after tDCS, delivered multiple stimulation sessions: two studies used five sessions (26, 27), two used 10 sessions (24, 29), one used 20 sessions (27) and two used 28 sessions (30, 43). Note that the latter two studies used HD-tDCS.

The only two single session studies that reported significant increases in patients' CRS-R scores were Thibaut et al. (23) and Naro et al. (25). The former assessed the effects of 20 min of 1 mA anodal tDCS over the L-DLPFC on 25 VS patients (6 traumatic and 19 nontraumatic) and 30 MCS patients (19 traumatic and 11 nontraumatic) and found that 43% of MCS patients showed some clinical improvements as measured by CRS-R. The same group effect was not found within VS patients, with only two patients showing post anodal tDCS-related improvements. A similar pattern was found in Naro et al. (25), where an improvement in CRS-R scores (in the motor and arousal sub-scales) was observed in MCS patients only, and not VS, after 10 min of anodal tDCS at 2 mA over the cerebellum.

More recent studies typically included multiple sessions with the aim of increasing the effects of stimulation. For example, Huang et al. (17) administered five daily sessions of anodal tDCS at 2 mA, for 20 min each, over the posterior parietal cortex in a sample of 33 MCS patients and found that 27% of them improved in their CRS-R scores. This included the appearance of new behaviors such as reproducible command-following, visual pursuit, intentional communication, object recognition, and intelligible verbalisation. While these represented a clear clinical improvement, the new behaviors remained consistent with a diagnosis of MCS in all cases. Moreover, these improvements were not present anymore at a 5-day follow-up. Other multiple session studies obtained similar results. For example, in Angelakis et al. (24), five sessions of anodal tDCS at 1 mA and five sessions at 2 mA, over the L-DLPFC or M1, led to CRS-R increases in all three MCS patients included in their study, and one VS patient out of 7. Similarly, Thibaut et al. (26), reported a two-point increase in CRS-R scores after five sessions of anodal L-DLPFC tDCS in a sample of chronic MCS patients (>5 months post injury). Interestingly, this effect was still present a week after receiving the last stimulation session. A further study including 20 tDCS sessions over the L-DLPFC (twice a day for 10 days) showed improvements in CRS-R scores in the MCS but not the VS patients assigned to the real stimulation group (27). In addition to these, Martens et al. (28), who also targeted the L-DLPFC, found a moderate effect size at the end of 4 weeks of 20 daily home-based tDCS sessions, although this effect only approached statistical significance. Using a different target (anodes on the left and right M1 and cathode on the nasion), Straudi et al. (29) found on average two points improvement in eight out of 10 chronic MCS patients (time post injury between 1 and 19 years), after 10 sessions of tDCS. Finally, the only two studies administering HD-tDCS, reported improvements in the CRS-R scores of all (six) MCS patients (43) and in 11 out of 20 MCS patients and in 5 out of 15 VS patients (30), after 28 sessions targeting the precuneus over 14 consecutive days. Collectively, these studies suggest that multiple tDCS sessions may be more effective than a single session in PDOC.

### Long Term Effects of tDCS

To date, we still have very little evidence in support (or otherwise) of a long-term therapeutic value of tDCS in this group. In fact, the above changes in CRS-R are typically registered shortly after stimulation (within 1 h). Only eight studies included follow-up measurements post-tDCS: at 12 months (23, 24), 3 months (28, 29, 40, 42), seven days (26), and 5 days (17). Among these, only Angelakis et al. (24) and Straudi et al. (29) found a lasting treatment effect. In the former, at 1-year follow-up, one out of seven VS patients progressed to MCS, while four VS patients remained stable as VS, and two VS patients died. In contrast two out of three MCS patients regained consciousness and one remained MCS. In the latter, eight out of 10 MCS patients showed an overall clinical improvement of two points in the total CRS-R score at 3 months post-tDCS, as compared to their first assessment. It is worth highlighting here that Straudi et al. (29), did not include sham or other control conditions, which limits the interpretation of their results. Overall, even though transient post stimulation effects are scientifically informative - in that they are suggestive of greater potential effects when stimulation protocols are refined - they would only have a real therapeutic value if they last days, weeks or months (45). Crucially, there is currently not enough evidence in support of long-lasting effects of tDCS even in healthy participants, or other clinical groups, when tDCS is administered at rest (that is, not coupled to a task). Indeed, most studies report effects that last a few minutes (46–48) to just a couple of hours (46, 49). This calls into question whether the above reported long term changes in CRS-R can indeed be attributed to tDCS or to the normal clinical progression of the patients.

In contrast, there is evidence that tDCS can lead to longer lasting effects when coupled to a task. For example, several studies in healthy volunteers have demonstrated increases in numerical performance or motor learning that can last days or even months post-stimulation (50–52). Rroji et al. (53) shed light on the mechanisms of these changes by showing that anodal tDCS over M1 coupled with motor practice (ballistic thumb flexion movements with the non-dominant hand) improved long-term (1 week follow-up) but not short-term retention (30 min and 1 day follow-up). This supports the idea that tDCS augments synaptic plasticity through long-term potentiation-like processes. In clinical groups, a pilot study on three chronic post-stroke aphasic patients showed that tDCS administered for 2 weeks (5 times per week) and coupled to naming training had beneficial effects in picture naming, that lasted up to 21 weeks after the end of stimulation (54). There are evident challenges associated with delivering tDCS during a task in PDOC, as many of these patients by definition cannot engage in purposeful behavior. However, due to the clear benefits of such an approach, this is an exciting avenue that could drastically improve the effects of tDCS in CMD patients who are able to voluntarily follow commands, albeit not behaviorally.

## Effects of tDCS on Brain Function

To date, no study has assessed tDCS changes using active fMRI/EEG paradigms. However, Zhang et al. (27) used EEG to investigate auditory event-related potentials (ERPs) changes in a passive paradigm (i.e., a task that does not require the patient's active participation) and found that tDCS increased P300 amplitude but not latency in MCS patients. Consistently with behavioral studies, the authors also observed that P300 waves appeared more frequently in MCS patients than in VS ones.

A much larger number of studies have focused on metrics derived from resting-state EEG recordings, such as power or different indexes of connectivity. For example, Naro et al. (25) assessed EEG connectivity of fronto-parietal networks (FPN) and found that anodal tDCS over the cerebellum modulated power in theta and gamma bands and increased coherence within the FPN in both VS and MCS patients. Importantly, these modulations correlated with increases in CRS-R scores only in MCS patients. Similar results were found by Bai et al. (34), who also assessed EEG coherence of the FPN and observed an increase in the theta band and a decrease in the gamma band coherence in MCS patients only after L-DLPFC tDCS. In a multi-session study, Cavinato et al. (41) found an increase in power and coherence of frontal and parietal alpha and beta bands in MCS patients following multiple anodal tDCS on the L-DLPFC. Interestingly, in VS patients tDCS led only to local changes in slow frequencies of anterior brain regions, suggesting a lack of integration of fronto-parietal functional networks.

More recently, Thibaut et al. (36) reported increased connectivity values in beta-2 EEG band (18–30 Hz) at the group level after multi-channel tDCS over left and right DLPFC. Importantly, they also identified higher relative power in theta band (4–8 Hz) and connectivity in beta band in tDCS responders (i.e., patients presenting a decrease in hypertonia in at least 2 joints after active but not sham tDCS) as opposed to non-responders. Similarly, Estraneo et al. (40) reported more normalized patterns in background EEG activity [according to the classification criteria proposed by (55)] in those patients whose CRS-R score improved after tDCS. Wu et al. (42) observed a trend toward higher EEG functional connectivity in frontal areas after 2 weeks of tDCS over the right and left DLPFC. More recently, Carrière et al. (37) assessed weighted phase-lag index (wPLI), symbolic mutual Information (wSMI), and EEG power bands changes following anodal tDCS over the L-DLPFC in 13 MCS patients. They observed higher wSMI connectivity between left and right parietal regions, and higher fronto-parietal wPLI connectivity, although these changes only appeared when uncorrected for multiple comparisons. Additionally, tDCS led to higher relative power at the group level in the alpha band (central regions) and theta band (frontal and posterior regions), but also at uncorrected statistics only. In their pilot study, Straudi et al. (29) found that multiple sessions of bilateral anodal tDCS over M1 led to modulations in resting-state EEG alpha band that correlated with improvements in consciousness. Lastly, a study that investigated changes in EEG relative band power and connectivity following multi-channel anodal tDCS over fronto-temporal areas found an increase in EEG complexity for 1-8 Hz bands (38). Moreover, increases in the difference between pre-tDCS CRS-R scores and post-tDCS CRS-R scores negatively correlated with EEG complexity for those bands.

While most EEG studies focused on connectivity and power band analyses, a small number of them assessed changes in cortical excitability instead. To this end, Bai et al. (33) combined transcranial magnetic stimulation (TMS) with EEG after delivering anodal tDCS over the L-DLPFC. They found that tDCS can indeed modulate cortical excitability in PDOC patients and identified differences in temporal and spatial domains between MCS and VS. Additionally, Naro et al. (32) used motor-evoked potentials (MEPs) to investigate the effect of anodal tDCS over the OFC and found boosts on cortical excitability in MCS patients and some VS patients.

Collectively, EEG studies suggest a trend toward increased functional connectivity following tDCS in MCS patients, which are more pronounced in patients who show behavioral responses to the stimulation. Interestingly, previous studies have associated recovery of consciousness with restoration of long-range connectivity [for a review see (56)]. It is thus possible that the reported changes in resting state EEG are indexing subclinical improvements, which suggests that further optimization of stimulation parameters may be needed to allow for these to translate into clinically measurable ones. However, as previously argued by Bestmann et al. (57), we do not yet have a good understanding of how the physiological effects of tDCS relate to its impact on behavior. This may delay the development of more effective treatments and promote mechanistic inferences that are not plausible.

## Common Methodological Limitations

A number of limitations could be explaining the variable success of tDCS in PDOC to date. While not aiming to provide an exhaustive list, some of the most important considerations are:

### Selection of Targets

As can be appreciated in our description above (see also Tables 1, 2), there is a high heterogeneity in the specific tDCS montage used across the different studies included in this review. This is likely due to the absence of a broadly accepted theory of consciousness that can explain the (full or partial) absence of awareness in PDOC (58, 59), and can provide a mechanistic framework for the selection of targets for stimulation.

This notwithstanding, most studies targeted the left DLPFC, and provide different justifications for this choice. For example, some selected this region on the basis of its role in integrating motor control and behavior, and its involvement in decision-making (27). Others refer to its connectivity with the default mode network and bilateral fronto-parietal networks (23) linked with self- and external awareness, respectively (60). Studies targeting the OFC allude to its role in translating integrated information into behavior (32), and those selecting the precuneus base this on its role in consciousness recovery (30, 43).

We have argued elsewhere (14) that the field needs better mechanistic models to inform the selection of targets for stimulation in PDOC. In the absence of a unified theory of consciousness, we have previously proposed to focus on external responsiveness instead by targeting motor areas (14). Some studies have targeted M1 or the cerebellum on the basis of their strong connections with the thalamus, and the central role of this structure in arousal regulation (27, 29). We suggest that thalamic modulations via motor regions would likely result in modulations of behavioral responsiveness instead. Indeed, we have provided evidence that tDCS over M1 and cerebellum can indeed modulate such connections in healthy volunteers and elicit neural changes associated with behavioral responses to commands (14).

It is important to note here that VS and MCS patients are characterized by different severity in their patterns of injury. Specifically, MCS patients show less severe white matter and thalamic damage (61, 62) as well as less extensive atrophies in basal ganglia (63). Many of the suggested mechanisms of action proposed to explain the selection of targets in PDOC rely on modulations of cortico-subcortical networks and therefore, these differences in damage could explain why tDCS appears to benefit only MCS patients.

### Electrode Placement and Inter-individual Anatomical Differences

Alongside challenges to identify a mechanistically informed target, there are also important considerations related to ensuring that the tDCS montage adequately targets the selected area in patients with very severe injuries and altered neuroanatomy. In line with most clinical tDCS research, studies in PDOC typically identify the location of the electrodes on the scalp by using EEG caps or manual measurements based on the 10–20 international electroencephalography system. While practical, this method is known to lack accuracy even in healthy volunteers, due to inter-subject brain variability. For example, in a recent study (64), the authors compared the localization of the DLPFC achieved with the 10–20 EEG system vs. MRI-guided neuronavigation and found that these varied significantly. These errors can only be expected to increase in PDOC patients, whose brains are characterized by gross abnormalities, such as brain atrophies, that are highly heterogeneous across patients, and lead to important anatomical and physiological changes (27). Seminal post-mortem studies showed that, while some abnormalities such as diffuse axonal injury or thalamic damage seem to appear in many patients, there was also high heterogeneity in the patterns of damage and the specific focal injuries observed (65, 66). It is thus possible that in many of the patients investigated to date, current was simply not reaching the target areas, or not at enough intensity.

Inter-individual anatomical differences are particularly important when using high-definition tDCS (HD-tDCS), an increasingly popular approach in the tDCS literature that we anticipate will quickly become popular in PDOC too. HD-tDCS can achieve higher spatial precision as compared to conventional tDCS, by circumscribing the current flow to the area of the small electrodes used (67). This method was designed to overcome limitations related to the poor spatial resolution of conventional tDCS, whereby due to the use of large electrodes, the electrical current being applied to the brain does not only reach the targeted area but also other surrounding regions (67). The diffuse nature of conventional tDCS makes it difficult to link observed effects (whether neural or behavioral) to the region originally being targeted and limits the effectiveness of tDCS for therapeutic uses. To our knowledge, only two studies assessed HD-tDCS in PDOC patients (30, 43), but we expect this to increase in coming years. However, it is important to note that as spatial specificity increases, so does the need of using robust and reliable current flow models to plan the location of the electrodes on the scalp to achieve an accurate targeting of the regions of interest (68). In fact, even in healthy volunteers, individual responses to tDCS can vary greatly due to differences in scalp and skull thickness or shape (69), cortical thickness, density or volume (70), or neurochemical factors (71). This has prompted the development of models that can be personalized to the individual anatomy of each participant (via structural MRI images) and can identify the best montage and doses to achieve the desired intensity in the target area (72). However, while recent years have seen a steep rise in the development of such models, and many are now available as part of commercial applications, these are based on healthy anatomy and tissue properties and have not been validated in severely injured brains. Although it is currently possible to use a patient's MRI data to create current models that take into account their specific neuroanatomy (and injury), in addition to focal injury, PDOC patients may have diffuse brain lesions that can greatly affect the microstructure of the remaining tissue and affect conductance.

The use of healthy models of current distribution to localize the best electrode placement for conventional and HD-tDCS may thus likely result in targeting the wrong anatomical region for many patients. In the specific case of HD-tDCS this can lead to even worse results than conventional (less focussed) tDCS. Therefore, we believe that the field needs the development of specific models that can incorporate individual differences in tissue abnormalities to aid the choice of montage and maximize the effects of tDCS across patients. We could further argue that such models are necessary before HD-tDCS can successfully be applied to PDOC patients.

In fact, it is only by gaining a deeper understanding of the observed variability in tDCS responsiveness - that is, understanding why tDCS is effective in some patients and not in others - that we can develop more effective, patient-focused protocols. Going forwards, the field needs studies to include neuroimaging methods within their protocols with the aim of identifying biomarkers that can help make predictions about responsiveness to stimulation protocols. In trials where the use of neuroimaging is not possible or feasible, we recommend a careful selection of candidate patients on the basis of absence of focal injuries in the target region(s) and nearby areas, as well as no gross abnormalities that result in region shifting. On the basis of the differences in subcortical damage discussed in section Selection of Targets above, we would argue that the selection criterion should go beyond focal cortical lesions to also include a relative preservation of the networks that tDCS intends to modulate with any given montage. While together, these requirements would clearly result in a reduced number of eligible patients, they would also result in better targeting and a better characterization of causality in the reported effects of tDCS. In our opinion, this is essential for the field to move forwards.

### Small Sample Sizes and Heterogeneity of Methods

As mentioned above, the majority of the studies in this review used small sample sizes; an average 20.7 (± 12.6) patients, that usually included both VS and MCS. In addition to this, patients' etiology is often varied with most studies including both traumatic and non-traumatic patients (for more details see Table 3). Moreover, as it is evident above, there is a great heterogeneity in the methods used (i.e., tDCS setups, number of sessions, outcome measures etc.). All together, these issues make comparisons across studies futile. While sample sizes and heterogenous patients are typical in the broader PDOC field, and inherent to conducting research with this very challenging clinical population, adopting more standard methods would facilitate meta-analyses, and perhaps the combination of data across multiple centers. This would allow us to achieve a better understanding of the variability in responsiveness to tDCS observed in PDOC patients.

**Table 3:** Demographics and etiologies of VS and MCS patients included in the different studies.

**References**	**VS patients' characteristics**	**VS patients' etiologies**	**MCS patients' characteristics**	**MCS patients' etiologies**	**Etiology of VS patients who showed clinical improvements**	**Etiology of MCS patients who showed clinical improvements**
**Single-session studies**						
Thibaut et al. (23)	N: 25; Gender: 9 females, 16 males; Mean age: 42 ± 17; BI onset: 24 ± 48 months.	6 TBI, 9 anoxic, 9 nontraumatic (5 CVA, 4 SH)	N: 30; Gender: 7 females, 23 males; Mean age: 43 ± 19; BI onset: 43 ± 63 months.	19 TBI, 4 anoxic, 6 non-traumatic (3 CVA, 3 SH, 1 mixed traumatic ischemic).	**2 VS patients:** both SH	**13 MCS patients:** 5 TBI, 4 anoxic, 2 CVA, 2 SH.
Naro et al. (32)	N: 12; Gender: 7 females, 5 males; Mean age: 54.4 ± 11.3; BI onset: 23± 21.5 months.	5 TBI, 7 anoxic.	N: 10; Gender: 5 females, 5 males; Mean age: 55.6 ± 15.9; BI onset: 13.9 ± 9.4 months.	5 TBI, 5 anoxic.	**4 VS patients:** all anoxic (add a note that these are only partially responders according to authors).	**All MCS patients:** 5 TBI, 5 Anoxic.
Naro et al. (25)	N: 10; Gender: 7 females, 3 males; Mean age: 51 ± 10; BI onset: 25 ± 24 months.	6 TBI, 4 anoxic.	N: 10; Gender: 5 females, 5 males; Mean age: 56 ± 17; BI onset: 14 ± 10 months.	3 TBI, 7 anoxic.	–	**All MCS patients**.
Bai et al. (33)	N: 9; Gender: 2 females, 7 males; Mean age: 45.8 ± 15.2; BI onset: 11.3 ± 6.8 months.	2 TBI, 4 anoxic, 2 haemorrhagic, 1 ischemic.	N: 7; Gender: 3 females, 4 males; Mean age: 47 ± 13.4; BI onset: 15.4 ± 11.5 months.	2 TBI, 3 anoxic, 2 hemorrhage.	–	–
Bai et al. (34)	N: 9; Gender: 2 females, 7 males; Mean age: 44.8 ± 14.5; BI onset: 11.4 ± 6.9 months.	3 TBI, 4 anoxic, 2 haemorrhagic.	N: 8; Gender: 4 females, 4 males; Mean age: 43.2 ± 14.9; BI onset: 12.6 ± 8.7 months.	4 TBI, 1 anoxic, 3 haemorrhagic.	–	–
Martens et al. (35)	N: 4; Gender: 1 female, 3 males; Mean age: 57.5 ± 19.6; BI onset: 3.1 ± 3.7 months.	1 TBI, 3 non-TBI.	N: 6; Gender: 1 female, 5 males; Mean age: 43.5 ± 20.8; BI onset: 8.9 ± 13.1 months.	5 TBI, 1 non-TBI.	**1 VS patient:** non-TBI.	**1 MCS patient:** 1 TBI.
Thibaut et al. (36)	N: 5; Gender: 3 females, 2 males; Mean age: 44.6 ± 12.1; BI onset: 9.4 ± 2.4 months.	3 TBI, 2 cardiac arrest	N: 7; Gender: 4 females, 3 males; Mean age: 38.5 ± 15.1; BI onset: 47.2 ± 35.5 months. 1 EMCS patient: male, aged 61, 10.6 months post BI.	3 TBI, 3 haemorrhagic stroke, 1 cardiac arrest EMCS patients: haemorrhagic stroke.	**1 VS patient:** Cardiac arrest	**2 MCS patients**: 1 TBI, 1 haemorrhagic stroke. **1 EMCS:** haemorrhagic stroke
Carrière et al. (37)	–	–	N: 11; Gender: 3 females, 8 males; Mean age: 46 ± 14; BI onset: 8.4 ± 6.3 months.	3 TBI, 3 cardiac arrest. 4 aneurysm, 1 meningitis.	–	**3 MCS patients:** 1 TBI, 1 cardiac arrest, 1 aneurysm.
Martens et al. (38)	N: 17; Gender: 9 females, 8 males; Mean age: 50.7 ± 12.8; BI onset: 7.7 ± 7.1 months.	2 TBI, 15 non-TBI.	N: 23 MCS; Gender: 6 females, 17 males; Mean age: 45.7 ± 15.9; BI onset: 61.8 ± 82.6 months. 6 EMCS patients: 3 females, aged 43.3 ± 16.3, 25 ± 24.8 months post BI.	12 TBI, 11 non-TBI. EMCS patients: all TBI.	–	**3 MCS patients:** 2 TBI, 1 non-TBI. **1 EMCS:** TBI
**Multiple session studies**						
Angelakis et al. (24)	N: 7; Gender: 2 females, 5 males; Mean age: 41.1 ± 12.5; BI onset: 62.4 ± 39.6.	3 TBI, 4 anoxic.	N: 3; Gender: 1 female, 2 males; Mean age: 39 ± 12.3BI onset: 21.3 ± 18.9 months.	2 TBI, 1 postoperative stroke.	**1 VS patient:** TBI	**All MCS patients:** 2 TBI, 1 postoperative stroke
Estraneo et al. (40)	N: 7; Gender: 3 females, 4 males; Mean age: 49.6 ± 24.5; BI onset: 13.8 ± 18 months.	1 TBI, 4 anoxic, 2 vascular.	N: 6; Gender: 3 females, 3 males; Mean age: 60.3 ± 13.2; BI onset: 27.16 ± 27 months.	2 anoxic, 4 vascular.	**2 VS patients:** 1 anoxic, 1 vascular.	**3 MCS patients:** 1 anoxic, 2 vascular.
Thibaut et al. (26)	–	–	N: 16; Gender: 7 females, 9 males; Mean age: 43.3 ± 15.15; BI onset: 78.25 ± 97.6 months.	11 TBI, 2 anoxic, 3 cardiac arrest	–	**9 MCS patients:** 5 TBI, 1 anoxic, 2 cardiac arrest.
Huang et al. (17)	–	–	N: 33; Gender: 13 females, 20 males. Mean age: 57 ± 11; BI onset: 6 ± 5 months.	20 TBI, 13 non-TBI.	–	No individual data provided
Zhang et al. (27)	N: 11; Gender: 6 females, 5 males; Mean age: 60 ± 17.5; BI onset: 4.5 ± 3.5 months.	4 TBI, 3 anoxic, 3 haemorrhagic	N: 15; Gender: 5 females, 10 males; Mean age: 42.7 ± 18.3; BI onset: 6.2 ± 4.4 months.	8 TBI, 2 ischemic, 4 haemorrhagic, 1 anoxic.	–	**8 MCS patients:** 4 TBI, 3 haemorrhagic, 1 ischemic.
Martens et al. (28)	–	–	N: 22; Gender: 6 females, 16 males; Mean age: 41.9 ± 12.5; BI onset: 106.3 ± 84.5 months.	10 TBI, 8 cardiac arrests, 3 aneurysm, 1 anoxic.	–	**6 MCS patients**.
Cavinato et al. (41)	N: 12; Gender: 5 females, 7 males; Mean age: 47.1 ± 16.2; BI onset: 80.28 ± 99.36 months.	2 TBI, 5 anoxic, 3 CVA, 3 SH.	N: 12; Gender: 5 females, 7 males; Mean age: 47.1 ± 16.2; BI onset: 64.2 ± 38.4 months.	6 TBI, 2 anoxic, 1 CVA, 1 SH.	–	Number of patients not specified.
Straudi et al. (29)	–	–	N: 10; Gender: 3 females, 7 males; Mean age: 35.4 ± 12.6; BI onset: 66 ± 64.8 months.	All TBI.	–	**8 MCS patients**.
Wu et al. (42)	N: 9; Gender: 3 females, 6 males; Mean age: 51.5 ± 11.1; BI onset: 102 ±61.5 months.	4 TBI, 1 anoxic, 4 haemorrhagic.	N: 7; Gender: 2 females, 5 males; Mean age: 42.6 ± 20.9; BI onset: 210.3 ± 189.6 months.	2 TBI, 2 anoxic, 3 haemorrhagic.	–	**1 MCS patient:** TBI
Guo et al. (43)	N: 5; Gender: -; Mean age: 49.9 ± 9.7; BI onset: 5.2 ± 1.9 months.	2 TBI, 3 haemorrhagic.	N: 6; Gender: -; Mean age: 55.5 ± 14.4; BI onset: 3.6 ± 1.1 months.	All haemorrhagic.	**3 VS patients:** 1 TBI, 2 haemorrhagic.	**All MCS patients**.
Zhang et al. (30)	N: 15; Gender: 3 females, 12 males; Mean Age; 51 ± 9.6; BI onset: 1.94 ± 3.42 months.	4 TBI, 11 haemorrhagic.	N: 20; Gender: 10 females, 10 males; Mean Age; 52.3 ± 16.9; BI onset: 3.3 ± 2.97 months.	4 TBI, 16 haemorrhagic.	**5 VS patients:** **1 TBI,** **4 haemorrhagic**.	**11 MCS patients:** **3 TBI,** **8 haemorrhagic**.

*BI, brain injury; CVA, cerebrovascular accident; EMCS, emerging from minimally conscious state; MCS, minimally conscious state; SH, subarachnoid hemorrhage; TBI, traumatic brain injury; VS, vegetative state*.

### tDCS Polarity and Control Condition

Due to constraints in resources and time availability, and other challenges that are also inherent to PDOC research, the majority of tDCS studies are focused on the effects of one polarity (typically anodal stimulation) and used sham to control for placebo effects. While the introduction of sham-controlled designs represented the first solid evidence in support of the effectiveness of tDCS, this approach is not without its limitations. Indeed, there is increasing evidence that current methods for sham stimulation do not always guarantee that participants and experimenters are unaware of the stimulation delivered [i.e., (70)]. More importantly, the lack of a control polarity or stimulation site limits the attribution of the resulting effects to the modulation of a particular brain region or network (used as target) instead of a broader generic effect over the brain or peripheral nerves (45). Although this may seem a scientific rather than clinical consideration, it can be argued that it is necessary to achieve a good understanding of the networks that modulate specific clinical effects in order to develop more effective stimulation protocols that can be targeted to the needs of specific patients.

Moreover, the choice of anodal stimulation as therapeutic is based on the assumption that this polarity has excitatory effects over neuronal populations, with cathodal tDCS typically having inhibitory effects. Following this assumption, *enhanced* or *reduced* behaviors are often explained by *increased* or *decreased* activity in specific cortical regions. Nevertheless, as uncovered by Bestmann et al. (57), this view might be over simplistic, as different cell morphologies and cortical folding of brain tissues have a profound influence on the net effects of stimulation.

### Challenges of Using Clinical Scales to Assess Responses to tDCS

It can be argued that for tDCS to have clinical significance as a therapeutic option it should lead to changes that can be assessed with current diagnostic scales (such as the CRS-R). However, it is also true that these scales are not sensitive to covert changes in cognitive functioning or awareness (73). On the one hand, clinical scales mainly rely on motor outputs, which can be highly inconsistent and limited in PDOC, due to lesions in motor cortices (15, 39, 74, 75). In addition, the presence of injury in language and visual cortices may also interfere with the patients' ability to understand the instructions and show appropriate responses (76). Furthermore, the presence of tracheostomy, altered reflexes, or arousal fluctuations (73, 76) adds further difficulties to detect clinical signs of awareness. In the most severe cases, the presence of CMD – whereby patients are by definition unable to produce motor behaviors, may mask such changes entirely. These difficulties are at the root of the high rates of misdiagnosis that are typically present in this population (76) and suggest that clinical assessments may not be well suited to reliably capture changes after tDCS.

It is thus possible that the challenges inherent to clinical assessments explain some of the inconsistencies in the above results. Recently developed clinical scales aimed at detecting subtle motor behaviors that may indicate residual cognition (and therefore a possible CMD) may offer a superior alternative to identify the effects of tDCS (39, 77). In addition, there is vast evidence that active fMRI and EEG paradigms (i.e., where patients are required to voluntarily follow commands) can successfully identify preserved but covert cognitive abilities and awareness in PDOC, in many instances before such changes become apparent clinically [see (78) for a review]. It is therefore possible that active electrophysiological/neuroimaging paradigms may be able to more accurately detect tDCS-induced effects on consciousness as compared to behavioral evaluations. However, while active tasks provide a definite answer that a patient can follow commands covertly and therefore are aware, they are also very cognitive demanding and may also fail to detect awareness in patients who are affected by language or working memory impairments. A recent study provided an alternative to active paradigms by focusing on the passive processing of the patients' peri-personal space; the multisensory-motor space that surrounds one's body (79). The authors found that certain EEG measures off peri-personal space were associated with preserved measures of neural complexity, suggesting a possible CMD. To further support this, peri-personal space metrics did not correlate with CRS-R (79). While confirmation of CMD in an individual patient requires a positive response in an active task, measures of peri-personal space offer a promising tool to detect sensorimotor changes mediating crucial self-environment interactions after tDCS.

### Challenges in Using Electrophysiological and Neuroimaging Studies

As can be appreciated in the summary above, the EEG metrics used across the different tDCS studies in PDOC is striking: three studies included coherence measures, six studies performed power spectral analyses (absolute power spectra or relative power spectra), one study used auditory ERPs, another used global mean field amplitude, a further study calculated the Lempel-Ziv-Welch complexity, and finally three investigated EEG connectivity at the electrode level using different connectivity measures (wPLI, wSMI and phase-locking value). This heterogeneity creates challenges when assessing whether results are consistent across studies or otherwise. Leaving the effects of tDCS aside, EEG assessments of awareness in PDOC have also been characterized by a complex variety of protocols, measures, and analysis pipelines (80), and beyond active tasks that can provide a proxy for covert command-following, the field has yet to agree on gold-standard measures of consciousness using EEG. While no study has used fMRI to characterize the effects of PDOC yet, it is important to highlight that the above considerations about heterogeneity of approaches and measures also apply to this technique (81).

It is also worth noting that the majority of EEG studies activity successfully identified changes in at least one of the measures considered, even when no significant changes in CRS-R were observed. Although this may speak for an increased sensitivity as a biological marker, we believe that it cannot currently be ruled out that false positives may partially explain the discrepancy between CRS-R and EEG findings in some of the studies included in this review instead. With this in mind, while we strongly believe that imaging and electrophysiology methods can shed light on the effectiveness of tDCS in low responsive patients such as those in PDOC, we also think that the field needs to work toward developing standardized outcome measures that can enable comparisons across studies.

Studies have used both active and passive tasks to successfully assess patients' residual consciousness. However, similarly to studies using EEG, neuroimaging studies also present a great deal of heterogeneity of paradigms used to evaluate consciousness. In addition to this, the majority of these studies included relatively small group of patients, and often lacked comparisons with healthy controls [for a metanalysis see (81)]. To further evaluate and support the diagnostic value of fMRI paradigms in PDOC patients, systematic replications of these studies are crucial.

## Integration of tDCS in Current Pathways of Care for PDOC

The pathway for care of PDOC patients proposed by the RCP (1) is composed of five different phases. This pathway is not linear, and patients can be moved up and down at any point during their care. For tDCS to make the jump from research studies into routine clinical care, it would need to be integrated into existing pathways. Importantly, several areas of study are still required before there is sufficient evidence to support the design effective tDCS interventions in PDOC, and we will review these in detail below. Notwithstanding this, here we review RPC pathways to provide a tentative framework to discuss the feasibility and potential uses for tDCS when integrated into current procedures.

The first phase refers to the acute care in the hospital ward and identifies as main priorities the confirmation of the origin of the disorder of consciousness (DOC) and the identification of the level of preservation of the primary neurological pathways. During this phase, if the DOC persists, the RCP suggests the following three steps, after 3 days, 2 weeks, and 4 weeks, respectively: assessment for interim advice, review and evaluation to eliminate treatable causes and, lastly, referral to specialist PDOC neurorehabilitation units. The second phase refers to the subacute/post-acute stage, which starts 4 weeks after injury (82) and involves early proactive management. In this phase the patient's DOC is considered prolonged (they are henceforth referred to as PDOC) and they are transferred to a specialist PDOC neurorehabilitation service for usually 2–4 months. During these, the patient's medical condition is stabilized, assessments of responsiveness are carried out and a care programme is set up. In the third phase, the patient is under continued active management with specialist PDOC monitoring. This phase lasts until the patient either recovers and is transferred to a specialist rehabilitation unit, or it becomes likely that they will remain in VS or MCS. In the latter case, their clinical status would be considered chronic (82). Importantly, the RCP (1) states that if the patient shows signs of change at this stage, they should be moved back to the second phase. The fourth phase (chronic PDOC) corresponds to long-term care, in the patient's own home or in a nursing home setting. The fifth and final phase refers to end-of-life care.

**Figure 2:**
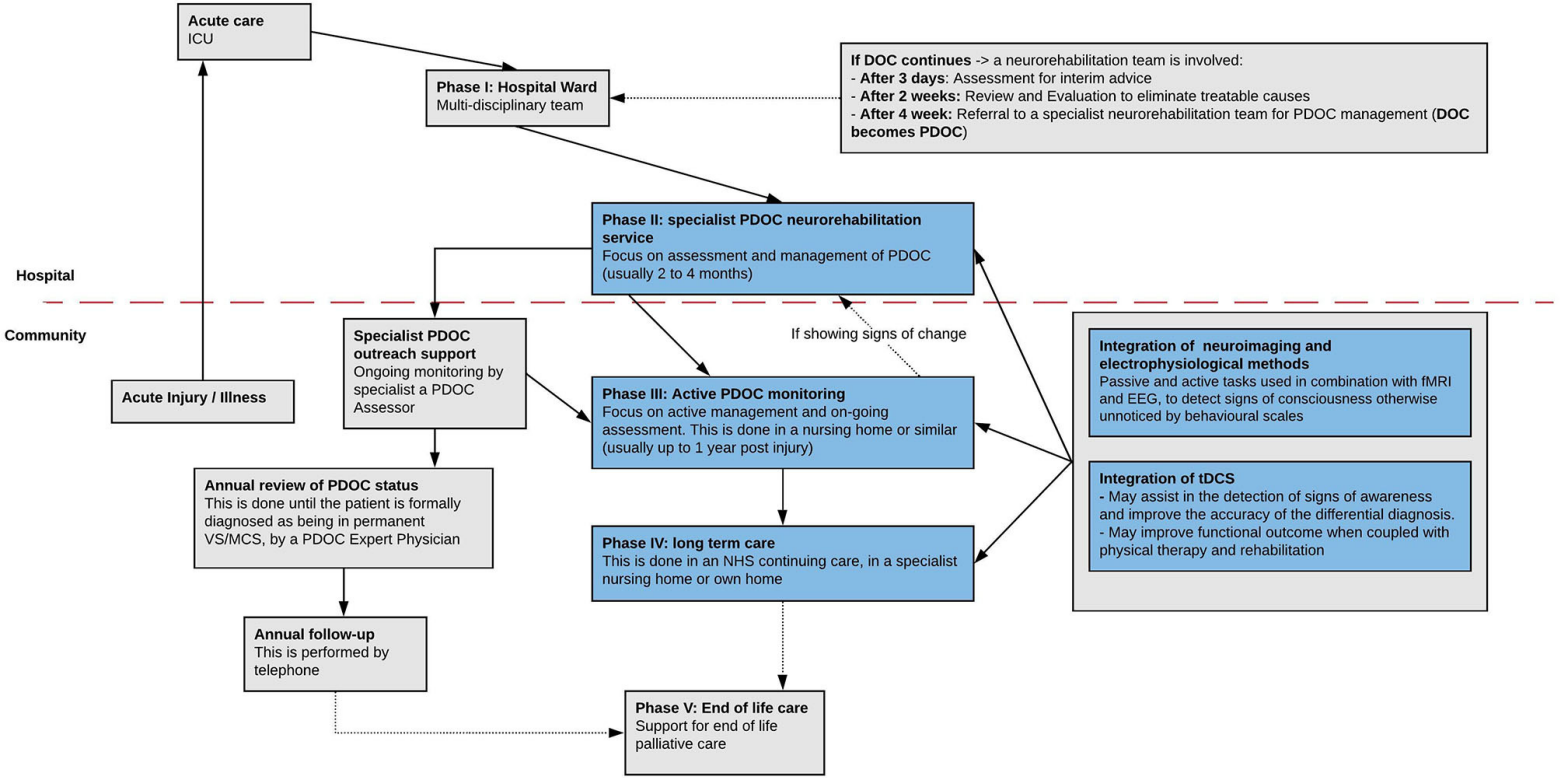
Representation of the pathways of care for PDOC patients as proposed by the Royal College of Physicians (1), with suggestions for the role that tDCS and neuroimaging/electrophysiological methods could play in each of the stages. Reproduced and amended from Royal College of Physicians (1).

To our knowledge, no study has explored the feasibility of delivering tDCS on DOC patients in the very acute setting (intensive care unit, ICU) or early stages of the subacute phase (phase 2). While administering tDCS at the ICU may not be possible, we argue that future studies should focus on the administration of tDCS it in the initial stages of subacute care (4 weeks after injury), to assess its potential for improving clinical diagnosis and assisting rehabilitation (Figure 2). By increasing patients' responsiveness, the clinical team would more easily recognize signs of awareness, and thus tDCS could help lower the chances of misdiagnosis as a result. Moreover, tDCS could be helpful in revealing covert signs of awareness even when motor behaviors are absent. For example, as reviewed above, Naro et al. (32) assessed residual connectivity in PDOC patients using EEG, and showed that tDCS over the OFC is able to uncover M1 excitability and premotor-motor connectivity in some VS patients, indicating they could potentially have CMD. While CMD can only be confirmed with neuroimaging methods, these are typically expensive and require experts to analyze and interpret the data. If tDCS can indeed identify signs of a potential CMD, this could be used to triage patients for referral for specialized neuroimaging studies. tDCS could therefore be a powerful tool for improving DOC differential diagnosis, when coupled with current behavioral scales (i.e., CRS-R) and electrophysiological methods. Decreasing the chance of misdiagnosis, either by improving detection of CMD in a patient or by differentiating between VS and MSC, is crucial for developing an adequate care plan. In fact, accurate assessment of consciousness has profound implications for patient care, as it is informative for prognosis and guides treatment decisions (such as end-of-life care decisions).

On the other hand, tDCS could improve the success of functional rehabilitation of PDOC in phases two and three. As mentioned above, tDCS is able to enhance motor learning in healthy participants and can aid in the recovery of motor functions in patients with brain lesions [for a review see (83)]. There is also evidence that concurrent brain stimulation and physical therapy can help motor rehabilitation [for a review see (84)]. In fact, according to Bolognini et al. (84), tDCS would augment the response of neural networks to motor training and, in turn, reinforce its long-term effects. Importantly, PDOC patients receive intensive physical therapy daily, as part of their management, from the second phase of care onwards. To our knowledge, there are no studies assessing the effects of combined tDCS and physical therapy in PDOC. Nevertheless, we can draw on the literature on other neurological diseases to assess the feasibility of using such methods during the early stage of treatment. For example, it has been observed that bi-hemispheric tDCS (anodal over the affected motor cortex and cathodal over the unaffected one) increases the gain of motor function induced by constraint-induced movement therapy in poststroke patients (85). Moreover, a study by Bornheim et al. (86) assessed the effects of repetitive tDCS (5 sessions) during the first month after stroke, while patients remained in the neurovascular unit, and observed that when administered prior to rehabilitation, tDCS led to clinically significant improvements in both functional motor outcomes and somatosensory functions. Similarly, Sattler et al. (87) showed that five consecutive daily sessions of anodal tDCS combined with radial nerve stimulation promotes motor hand recovery in the acute phase after ischemic stroke in the rehabilitation unit. Importantly, patients included in this study had their stroke within a maximum of 2 weeks before starting stimulation. With a patient group more directly related to PDOC (moderate to severe traumatic brain injuries, TBI), Ulam et al. (88) assessed cumulative effects of 10 anodal tDCS sessions on both EEG oscillations and neuropsychological tests while patients were undergoing neurorehabilitation during the acute to subacute stages (~7 ± 4 weeks). Although no cognitive gains were observed, tDCS successfully induced changes in brain oscillations in several frequency bands that suggested an increase in cortical excitability in such patients. Importantly, these changes correlated with improvements in performance.

Taken together, tDCS studies in stroke and TBI, demonstrated that tDCS can accelerate and improve functional recovery while paired with conventional rehabilitation. Moreover, they proved the safety and feasibility of administering repetitive tDCS in the subacute stages after brain injury. We believe this is an exciting area for development in future PDOC studies. The field urgently needs evidence that tDCS can indeed add to the effects of routine clinical interventions. Literature on stroke and TBI suggests this is a research avenue worth pursuing. In addition to these earlier stages, the beneficial effects of tDCS could be investigated also in combination with the routine care during of the fourth phase (the long-term care, or chronic stage) at patients' homes, rehabilitation centers or nursing homes. Martens et al. (28) showed that tDCS can be appropriately used by patients' relatives and caregivers after proper training and can lead to some clinical improvements that may have an impact on the quality of life of patients or caregivers. While these are promising results, and in our opinion offer strong support for the feasibility of this approach, the field still lacks evidence in support of long-term effects even after repeated sessions at home. As we have argued in earlier sections, tDCS is particularly well suited for home delivery due to its portability and safety. Therefore, we think that further research should focus on this stage, where patients would not typically receive any other intervention, to corroborate whether tDCS can offer an alternative to improve their quality of life.

## Conclusions

In conclusion, there is consistent evidence that tDCS can lead to neural changes and measurable clinical improvements in a subset of MCS patients. Moreover, research to date suggests that the use of multiple stimulation sessions can further enhance the clinical effects of tDCS in this group (with 2 single-session vs. six multiple-session studies leading to changes in CRS-R score). However, research has been highly heterogeneous in their specific choice of stimulation parameters, montages, protocols, and outcome measures, and it is yet not possible to draw any conclusions about what factors may explain why some patients respond and others do not as a result. In addition to this, studies have typically used relatively small sample sizes. While it is important to recognize that achieving large sample sizes of PDOC patients in single centers is a challenge, we should also acknowledge that tDCS is well-known for leading to highly heterogeneous responses and very large samples are required to obtain reliable and reproducible effects. We therefore think that the field urgently needs multi-center studies that are appropriately powered to assess the effect of individual differences in the responses elicited by tDCS in PDOC. Crucially, these studies should include appropriate assessments to capture potential long-term effects of tDCS, which are required for any intervention to be clinically useful.

Furthermore, we have argued that the use of electrophysiological and neuroimaging techniques, alongside conventional behavioral assessments of consciousness may identify covert changes after stimulation but may also support the characterization of inter-individual differences, and, in turn, lead to the development of more patient-specific stimulation protocols. Specifically, the field needs the development of current flow models that consider patients' specific neuroanatomy and lesions and can be used to find the best electrode placement to achieve the desired effects in each individual patient. Such models would also be crucial to stratify patients who are more likely to respond to tDCS. We argue that this step of individualizing treatments is imperative to the application of tDCS in clinical settings.

Finally, there is robust evidence that tDCS can lead to much stronger and long-lasting effects when paired with a task that can engage the networks of interest. We believe that this supports its integration into existing rehabilitation programmes and physical therapy to exploit potential synergies, and that studying whether such synergies exist should be a priority for future studies. Should this be demonstrated, we argue that its implementation is feasible from the second phase of the national pathways of care recommended by the RCP (i.e., subacute stage of rehabilitation), where patients have the greatest potential for improvement. In addition to this, we believe it is crucial to assess the role of tDCS in the chronic phases of injury with a focus on improving patients' quality of life.

## Author Contributions

DA, AR, and SC performed literature research. DA and DF-E wrote the manuscript. All authors critically reviewed the manuscript and contributed to the editing of the final draft.

## Conflict of Interest

The authors declare that the research was conducted in the absence of any commercial or financial relationships that could be construed as a potential conflict of interest.
